# The influence of COVID-19 pandemic on the mental health of pharmacists as frontline health care providers in Nepal

**DOI:** 10.1016/j.heliyon.2024.e29132

**Published:** 2024-04-03

**Authors:** Subesha Adhikari, Sarina Pradhan Kasaju, Uma Langkulsen

**Affiliations:** Faculty of Public Health, Thammasat University, Pathum Thani, 12120, Thailand

**Keywords:** Burnout, Exhaustion, Disengagement, Wellbeing, Nepal

## Abstract

**Background:**

Due to COVID-19, pharmacists have been exposed to a variety of dangers that have an impact on their mental health. The study highlights that impact of COVID-19 and work led to burnout among them. Thus, it is necessary to offer mental health services.

**Aims:**

To assess the impact of COVID-19 on the work of pharmacy professionals and ascertain the extent of the influence of burnout on mental health among pharmacists working as frontline health providers in Nepal.

**Methods:**

Pharmacists from Province 2, 3, and 5 in Nepal participated in a self-administered questionnaire that assessed mental health and wellbeing and burnout using 16-item OLBI. Data was collected from January 2023–March 2023. Independent sample *t*-test, one-way ANOVA, Pearson's correlation, and linear regression were employed to identify any significant connections between burnout and mental health and wellbeing.

**Results:**

Out of 243 participants**,** COVID-19 pandemic and work was found to have a negative impact on the mental health and wellbeing of a 33.7% of pharmacists. Participants reported having financial issues in 41.6% of cases and 9.5% considered leaving the profession and were concerned about the provision of service quality and making mistakes at work. A strong and meaningful positive relationship and linear regression were observed between exhaustion, disengagement, burnout, mental health and wellbeing. Pharmacists working part-time (p < 0.050) in hospital settings experienced burnout as a result of insufficient training, extended working hours, and stress both on and off the job.

**Conclusions:**

Pharmacists are to prioritize a healthy work-life balance, which includes avoiding prolonged shifts >8 h, regular physical exercise and promote open communication among colleagues to address workplace concerns.

## 1 Introduction

The Severe Acute Respiratory Syndrome Coronavirus 2 (SARS-CoV-2) continues to pose a global threat to public health. The initial instance of Coronavirus (COVID-19) was identified in Wuhan, China in December 2019, and the World Health Organization (WHO) declared it a pandemic in March 2020 [1]. The COVID-19 pandemic has had a profound impact on the mental health and wellbeing of people worldwide. Among those significantly affected are healthcare workers (HCWs), who face heightened psychological stressors due to their visible presence on the frontlines. These stressors can have lasting effects on the wellbeing and mental health of HCWs and also impact their perception of the value and safety of the care they deliver [1]. Glancing at the global contexts, HCWs including pharmacists of China, Italy, Saudi Arabia, the Dominican Republic, Iran, Pakistan, India and Nepal have described COVID-19's adverse psychological impacts including stress, fear, anxiety, burnout and mental tiredness [2,3]. Pharmacists experience significant mental burden while providing care to patients, which has a detrimental impact on their psychological health. The prevalence of burnout in the pharmacy industry has gained recognition, as numerous studies have demonstrated substantial impact of work-related burnouts and stress on pharmacists [4]. In the United States, a significant proportion of pharmacists face excessive workloads and job stress, with more than half experiencing high levels of workload burden and 61.2% pharmacists reporting burnout in the workplace. When compared to their healthcare providers in hospital settings, pharmacists reported higher levels of mental distress during the COVID-19 pandemic [5]. In Canada, 85% of community pharmacy professionals reported worsened mental health due to COVID-19. Younger and hourly-paid professionals had higher turnover intentions. Those with dynamic schedules or employed by large pharmacy chains had lower mental health quality [6]. A study in Swedish community pharmacists showed that during the pandemic, 62% of pharmacists perceived an increased workload. Additionally, 47% found the physical work environment worse, and 59% noted a deterioration in the psychosocial work environment [7]. Similarly, a study conducted in Portugal affirmed that pharmacists lacked confidence in delivering services to consumers, which had repercussions on their mental health and wellbeing [8].

In Japan, Serbia, and India, pharmacists experienced elevated levels of anxiety, depression, and burnout due to factors such as lockdown measures, business instability, and continuous interaction with the community [9]. A study in Indonesia, showed that 41.1% of health workers including pharmacists were more stressful while serving the public during COVID-19. They found out that 34.3% of female health workers experienced stress, 36.7% experienced post-traumatic stress and 40.1% of both the male and female workers had moderate levels of depression [2].

A significant proportion of pharmacists in Wuhan, specifically 60.3%, reported experiencing moderate to high levels of anxiety and high stress. This percentage is higher when compared to surgeons, oncologists, and emergency physicians [5]. The impact of COVID-19 on HCWs is comparable to the stress experienced during natural disasters or large-scale conflicts. In a study conducted in Pakistan, it was found that a great majority of HCWs, including pharmacists, experienced signs of despair and nervousness due to the COVID-19 pandemic [10].

In Nepal, the initial case of COVID-19 was detected in January 2020. Even though the COVID-19 pandemic had largely subsided by January 2023 (1,001,002 cases), the fact that by March 2023 there were 1,001,325 cases, indicates that the COVID-19 pandemic was not entirely over during the data gathering phase [11].

Pharmacists in the country have encountered stress and anxiety due to fluctuations in business and the responsibility of addressing even minor symptoms like cough and cold of patients who do not prefer visiting doctors directly [12]. Pharmacists have been active in ensuring the timely supply of medicines, conducting screenings for suspected cases, and promptly referring individuals to hospitals when necessary. Their efforts have not only supported government initiatives in the pandemic preparedness but also stimulated constructive thinking for planning effective strategies to manage the situation more commendably [13]. Amidst the COVID-19 pandemic, a significant proportion (23.6%) of pharmacists in Nepal exhibited indications of borderline anxiety, while 37.5% reported experiencing symptoms of depression. Additionally, a few pharmacists faced challenges with insomnia (34%) and reported instances of burnouts [14]. The mental wellbeing of pharmacists in Nepal has been significantly impacted by the COVID-19 pandemic and their work responsibilities. The study highlights the psychological distress experienced by pharmacists, with 18.8% of them being forced to close their businesses, leading to heightened levels of mental agitation [12]. A recent study conducted in Chitwan, Nepal during the second wave of COVID-19 pandemic showed 77.9% were severely distressed while 22.1% were mild to moderately distressed as a result of the COVID-19 pandemic and the impact of their work [15].

The study emphasizes the importance of identifying and addressing pharmacist's wellbeing by drawing attention to the effects of the COVID-19 pandemic and work on their mental health. It emphasizes the value of encouraging work-life harmony, encouraging collaborative care, and putting in place mental health-focused policies. Given the scarcity of studies that reveals the association between burnout and mental health and wellbeing of pharmacists in Nepal, this issue holds significant importance. Despite a few mental health studies conducted in Nepal [13,14,16] among healthcare workers, including pharmacists, these studies have not specifically addressed the challenges faced by part-time employees and undergraduate students who may experience disengagement and exhaustion due to the demands of work during the COVID-19 pandemic. This study aims to address this gap in the literature.

**Research Question:** What is the influence of the COVID-19 pandemic on the mental health of pharmacists as frontline health providers in Nepal?

## 2 Methods and materials

### 2.1 Study area

**Fig. 1:**
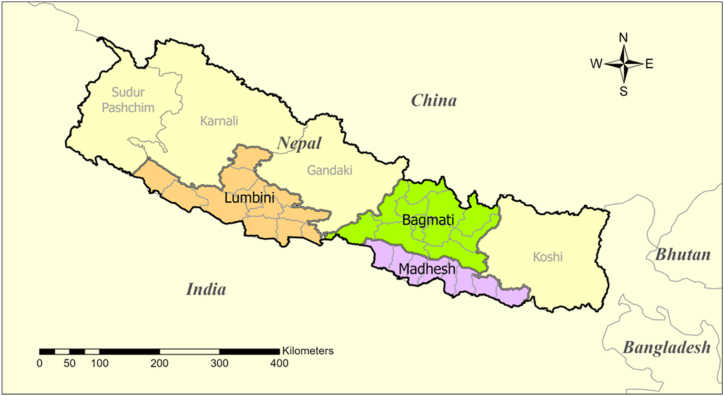
Study area.

A descriptive cross-sectional quantitative study design was used wherein all variables were assessed at a single point of time. The data collection period spanned from 2 January 2023 to 15 March 2023. The study was conducted in three provinces of Nepal, namely Madhesh (Province 2), Bagmati (Province 3), and Lumbini (Province 5) as shown in Fig. 1. The three provinces were purposely chosen as they have digital infrastructure of over 2000 allopathic pharmacies and having one every 2 km from wherever a person is [17].

### 2.2 Data collection

The study comprised pharmacists who met specific criteria, including possessing a minimum bachelor's degree in pharmacy or an equivalent qualification from a recognized educational institution. The study population consisted of pharmacists working in both government and private hospitals, proficient in English and Nepalese languages, aged 18 or above, and having a professional experience of 1–3 years since the onset of the COVID-19 pandemic. Pharmacists specializing in Ayurvedic or homeopathic practices were not included in the study. A total of 243 subjects were selected through stratified sampling, which involved randomly selecting allopathic pharmacies from public and private hospitals, as well as other community pharmacies, across three provinces. The findings highlight the complicated nature of the challenges faced by pharmacists in Nepal during the COVID-19 pandemic and emphasize the critical need for targeted support and interventions to safeguard their mental health and wellbeing as they continue to serve on the frontline of healthcare provision.

A self-administered questionnaire was designed and used to collect data for the study. The first and the second section of the questionnaire consisted of socio-demographic items and mental health and wellbeing items which were adopted from a former study where questions were designed especially for pharmacist workforce [18]. The third section was Oldenburg Burnout Inventory (OLBI), it included both negatively and positively worded questions for each area, lowering the danger of decorated truthful association and simultaneous response biases [19], hence making it a better psychometric scale [20]. The OLBI has been approved for usage in a variety of populations and environments [21]. OLBI comprises of 16 items of positive and negative worded questions that cover 2 scales; disengagement (OLBI-DE, 8 items), and exhaustion (OLBI-E, 8 items) with Cronbach's alpha- 0.70 and 0.75 respectively [22]. After pilot testing among 30 subjects in Nepal, the Cronbach's alpha for disengagement and exhaustion was 0.71 and 0.74 respectively and the average Cronbach's alpha of the OLBI items were found to be 0.725.

### 2.3 Data analysis

Socio-demographic characteristics were analyzed using descriptive statistics. The mean values of the high and low risk burnout groups were compared using the independent sample *t*-test and one-way ANOVA. To examine the association between OLBI and mental health and wellbeing, linear regression and bivariate correlation analyses were conducted. In statistical terms, a significance level was defined as a p < 0.050. Data analysis was performed using International Business Machines (IBM) Corporation's globally recognized software, Statistical Package for Social Sciences (SPSS) version 25.

## 3 Results

### 3.1 Demographic characteristics of the subjects

Table 1 presents the socio-demographic characteristics of the 243 subjects. It was found that 49.8% of the subjects were in the age group of 26–35, while only 2.1% were above the age of 55. In terms of gender distribution, 45.7% were female and 54.3% were male. The majority of subjects (52.3%) had a Bachelor's degree in Pharmacy, and 41.6% were employed in community settings. Around 37.9% worked in hospital areas, while only 11.1%, 5.8%, and 3.7% practiced in pharmaceutical industries, primary care, and academic/educational institutions, respectively. Notably, 19.8% of the subjects had been working as pharmacists for over 10 years, and 68.7% were employed on a full-time basis.

**Table 1:** Exploring mental health and work challenges: socio-demographics, wellbeing, career intentions, and service quality.

Variables	Frequency (%)
Socio-demographic characteristics (N = 243)	n (%)
Age	
≤25	61 (25.1)
26–35	121(49.8)
36–45	48(19.8)
46–55	8(3.3)
≥56	5(2.1)
Sex	
Female	111(45.7)
Male	132(54.3)
Ethnicity	
Brahmin	97 (39.9)
Chettri	51 (21.0)
Janjati	77 (31.7)
Ethnic minorities	16 (6.6)
Others	2 (0.8)
Religion	
Hindu	201 (82.7)
Buddhist	25 (10.3)
Christian	7 (2.9)
Muslim	9 (3.7)
Others	1 (0.4)
Education Qualification	
Diploma	23 (9.5)
B. Pharm	127 (52.3)
Post-graduate Pharmacy Fellowship	6 (2.5)
MSc./M Pharm	19 (7.8)
Pharm D	65 (26.7)
Ph D	3 (1.2)
Area of practice	
Community	101 (41.6)
Hospital	92 (37.9)
Academic/Educational body	9 (3.7)
Pharmaceutical industry	27 (11.1)
Primary care	14 (5.8)
Career stage	
<5years	119(49.0)
5–10 years	76(31.3)
>10 years	48(19.8)
Employment type	
Part-time	76(31.3)
Full-time	167(68.7)
Marital status	
Single	120 (49.4)
Married	119 (49.0)
Divorced	1 (0.4)
Separated	1 (0.4)
Widowed	2 (0.8)
Family Type	
Nuclear	110 (45.3)
Joint	129 (53.1)
Extended	4 (1.6)
**Multiple reasons behind negative impact on mental health and wellbeing**	**n (%)**
Financial problems	101(41.6%)
Concerns of contacting COVID-19 or transmitting it to others	74(30.5)
Lack of work-life balance	69(28.4)
Stress at work	58(23.9)
Stress outside work	38(15.6)
Long working hours	37(15.2)
Illness	55(22.6)
**Consideration on leaving the profession**	**n (%)**
Yes, I have considered leaving the pharmacy profession	23(9.5)
No, I have not considered this	167(68.7)
Don't know/Not applicable	53(21.8)
**Service quality concerns**	**n (%)**
Always	45(18.5)
Often	39(16)
Occasionally/Sometimes	118(48.6)
Never	41(16.5)
**Concerns about making mistakes at work**	**n (%)**
Always	27(11.1)
Often	30(12.3)
Occasionally/Sometimes	131(53.9)
Never	55(22.6)

### 3.2 Multiple factors contributing to the negative impact on mental health and wellbeing

Table 1 summarizes reasons contributing to the negative impact on the mental health and wellbeing of pharmacists in Nepal who have been at the forefront of healthcare provision during the COVID-19 pandemic. Out of the 243 subjects surveyed, significant proportions reported various challenges that affected their psychological health and wellbeing. Financial problems emerged as a prominent concern, with 41.6% of subjects testifying to their adverse impact. The burden of financial difficulties weighed heavily on their overall mental health. Additionally, 30.5% expressed anxiety about contracting COVID-19 or transmitting it to others, leading to heightened stress levels and a subsequent decline in mental wellbeing.

Long working hours, according to 15.2% of the subjects exacerbated the strain on their mental health and wellbeing. The demands of studying and training also surfaced as sources of distress, affecting the mental health of 14.8% of pharmacists amidst the pandemic. This suggests that the additional responsibilities and requirements placed upon them during this crisis had a profound impact on their overall psychological wellbeing. Furthermore, inadequate staffing levels contributed to the mental health burden, with 13.2% of subjects expressing distress due to the lack of support. Additionally, 11.5% highlighted lack of appropriate remuneration despite serving as frontline health providers during the COVID-19 pandemic, further exacerbating the strain on their mental health and wellbeing. Remarkably, 7.4% of the subjects faced the added challenge of being unable to find full-time work, compounding their mental health struggles and paralleling their fight against the COVID-19 pandemic.

***Pharmacists' Wellbeing and Work Challenges amidst COVID-19***Table 1 also provides insights into the experiences of pharmacists during the COVID-19 pandemic, specifically regarding considerations of leaving their profession regarding concerns about service quality, and worries about making mistakes at work due to the impact on their mental health and wellbeing. Out of the total subjects, 9.5% (23 pharmacists) expressed thoughts of leaving the pharmacy career. The majority, comprising 48.6% (118 pharmacists), reported occasional/sometimes concerns about the quality of service they provide, while 53.9% expressed occasional/sometimes concerns about making mistakes at work. Furthermore, 12.3% (30 pharmacists) reported frequent worry about making mistakes at work, and 11.1% (27 pharmacists) were consistently concerned about this issue. Exhaustion and disengagement at the end of a typical working day was reported by 38.3% (93 pharmacists) and 10.3% (25 pharmacists) respectively. Additionally, 9.9% indicated a desire to take sick leave but felt unable to do so. Regarding the overall assessment of mental health and wellbeing, among the 243 subjects, 37.4% (91 pharmacists) rated their state as ‘okay,’ while 32.5% (79 pharmacists) rated it as ‘not good,’ whereas the rest of them rated their state as ‘good’. Out of the total pharmacists surveyed, 33.7% (82 pharmacists), reported a negative impact on their mental health and wellbeing due to work during the COVID-19 pandemic. Meanwhile, 33.3% (81 pharmacists), stated that their work had neither a positive nor negative impact, and 11.5% were uncertain about the impact on their mental health. The remaining pharmacists reported a positive impact on their mental health while working as frontline health providers in Nepal.

### 3.3 Socio-demographic correlates, exhaustion, and disengagement in pharmacists during COVID-19

A bivariate correlation analysis was conducted to examine the linear relationship between socio-demographic variables and burnout, represented by the average of exhaustion and disengagement, as well as exhaustion and disengagement as separate variables. The results, detailed in Table 2, indicate statistically significant relationships (p < 0.050) with burnout. Religion, employment type, and marital status emerged as significant factors. A positive correlation was observed between respondents' religion and burnout (r = 0.10, p = 0.040), suggesting a mild association. Conversely, a negative correlation was identified between employment type and burnout (r = −0.18, p = 0.004), indicating that different employment types are linked to varying levels of burnout. Similarly, marital status showed a negative correlation with burnout (r = −0.20, p = 0.002), emphasizing that the marital status of respondents is associated with the experience of burnout.

**Table 2:** Correlation between Socio-demographic variables and Burnout.

Independent Variables	BURNOUT		EXHAUSTION		DISENGAGEMENT	
	Pearson Correlation (r)	P-value	Pearson Correlation (r)	P-value	Pearson Correlation (r)	P-value
Age	−0.11	0.07	−0.12	0.05	−0.08	0.18
Sex	−0.01	0.76	−0.03	0.61	−0.001	0.98
Ethnicity	0.03	0.63	−0.006	0.92	0.06	0.31
Religion	0.13	0.04*	0.11	0.06	0.12	0.05
Education Qualification	−0.008	0.89	0.001	0.98	−0.01	0.78
Area of practice	0.11	0.07	0.01	0.77	0.20	0.002*
Career stage	−0.08	0.16	−0.08	0.19	−0.08	0.21
Employment type	−0.18	0.004*	−0.15	0.01*	−0.18	0.004*
Marital status	−0.20	0.002*	−0.20	0.002*	−0.16	0.008
Family type	0.006	0.92	−0.29	0.65	0.04	0.50

P-value = Probability value.

*p < 0.050.

The table highlights the associations between socio-demographic characteristics of pharmacists working as frontline health providers during the COVID-19 pandemic in Nepal and exhaustion as a dependent variable. Significantly, employment type and marital status exhibit a statistically significant relationship with exhaustion (p < 0.050). Despite the statistical significance, both employment type and marital status show a negative correlation with exhaustion. Employment type has a correlation coefficient of −0.15 (p = 0.010), indicating that different employment types are associated with varying levels of exhaustion. Similarly, marital status demonstrates a correlation coefficient of −0.20 (p = 0.002), suggesting an association between marital status and the experience of exhaustion.

The table reveals significant associations (p < 0.050) between socio-demographic variables (area of practice, employment type, marital status) and disengagement. Positive correlations exist for area of practice (r = 0.20, p = 0.002), while negative correlations are noted for employment type (r = −0.18, p = 0.004) and marital status (r = −0.16, p = 0.008) with disengagement. However, no statistically significant relationships (p > 0.050) were found between age, sex, ethnicity, education qualification, career stage, and family type with burnout, exhaustion, and disengagement, indicating an absence of correlation between these variables.

Using one-way ANOVA the association between socio-demographic variables like: age, ethnicity, education qualification, area of practice, career stage, marital status, family type and mental health and wellbeing did not differ significantly (p > 0.050). However, the association between religion and psychological health and wellbeing differed significantly (p < 0.050). In addition, one-way ANOVA was used to detect the association between sociodemographic variables: age, ethnicity, religion, career stage, family type and burnout. This was not statistically significant (p > 0.050). On the other hand, the association between education qualification, area of practice, marital status and burnout was found to be statistically significant (p < 0.050) which specified pharmacists having burnout. Independent *t*-test was used to detect the association between sex and employment type with mental health and wellbeing and burnout where the first association did not show significant difference but the second one showed difference signifying burnout.

### 3.4 Unveiling the threads: correlation and regression analysis of exhaustion, disengagement, burnout, mental health, and wellbeing

Table 3 illustrates a statistically significant relationship (p < 0.050) between exhaustion, disengagement, burnout, and mental health and wellbeing of pharmacists. A strong positive correlation was observed between exhaustion (r = 0.31, p < 0.001), disengagement (r = 0.27, p < 0.001), burnout (r = 0.32, p < 0.001), and mental health and wellbeing of pharmacists. The R squared values of 0.10, 0.07, and 0.10 for exhaustion, disengagement, and burnout, respectively, indicate 10% and 7% variance in mental health and wellbeing. The B-coefficients demonstrate a significant and positive impact of exhaustion (B = 0.33, p < 0.001), disengagement (B = 0.32, p < 001), and burnout (B = 0.39, p < 0.001) on the mental health and wellbeing of pharmacists working as frontline health care providers in Nepal during the COVID-19 pandemic.

**Table 3:** Correlation and regression between exhaustion, disengagement, burnout and mental health and wellbeing.

Independent Variables	Pearson Correlation (r)	P-value	R squared	B coefficient	P-value
**Exhaustion**	0.31	0.000*	0.10	0.33	0.000*
**Disengagement**	0.27	0.000*	0.07	0.32	0.000*
**OLBI (Burnout)**	0.32	0.000*	0.10	0.39	0.000*

OLBI= Oldenburg Burnout Inventory.

R squared = Coefficient of Determination.

B coefficient = Beta coefficient.

P-value = Probability value.

*p < 0.050.

## 4 Discussion

A significant proportion of the subjects indicated that their overall mental health and wellbeing were negatively affected, experiencing challenges in various aspects of life such as work, home, and interpersonal relationships due to the impact of COVID-19 and their professional responsibilities. Pharmacists have played a crucial role since the start of the pandemic, including activities such as raising awareness and referring potential cases of infection [23]. In this study, a considerable proportion of subjects did not utilize any sick leave, however 9.9% expressed their desire to take sick leave but felt unable to do so, and this was significantly correlated with burnout. Moreover, a substantial number of subjects reported contemplating leaving the pharmacy profession. Additionally, subjects who did not experience enjoyment in their work as pharmacists, particularly due to the influence of COVID-19 and their professional responsibilities, also exhibited a significant correlation with burnout. Subjects in the study expressed occasional concerns about the quality of services they provide and occasional worries about making mistakes at work. The disruption of work-life balance emerged as a pressing issue for 28.4% of subjects, highlighting the challenges faced in managing personal and professional responsibilities amid the pandemic. Furthermore, 23.9% and 22.6% of subjects identified work-related stress and illness as significant factors negatively impacting their mental health and wellbeing, underscoring the toll of the pandemic on their overall psychological resilience. Beyond the workplace, stressors outside of work impacted 15.6% of subjects, indicating the complex interplay between personal and professional stressors during these challenging times.

In this research project, a significant proportion of subjects (41.6%) reported experiencing financial difficulties, which was followed by concerns regarding contracting and transmitting COVID-19 to others. This finding aligns with the results of a study conducted among pharmacists in Nigeria [4]. The adverse effects of work on pharmacists result in dissatisfaction and potential turnover, ultimately impacting their mental health and potentially leading to the development of a negative attitude [24]. Pharmacists always feared transmitting the disease to other staff in the workplace along with the residents [25]. The study revealed that a significant number of pharmacists experience burnout, and this finding is consistent with a survey conducted in the United Kingdom, which also identified a great jeopardy of burnout among pharmacists [18]. In addition, pharmacists were found to be disengaged after work as a result of burnout [8]. Furthermore, healthcare workers in the United States reported a high risk of burnout during the COVID-19 pandemic, indicating a similar trend [26].

The experience of burnout has led nearly half of the pharmacists to have feelings of depression and or hopelessness, as well as a sense of dissatisfaction when things are not done perfectly or according to specific expectations. As a result, there is an urgent need for mental health support for pharmacists as well as other health professionals, particularly for those in developing countries where they are inadequate as burnout among pharmacists and loss in productivity [27], have been determined to be of a great propensity to leave one's current practice while still actively employed [4]. The present study differs from previous research piloted in Nepal as this study not only detected the impact of COVID-19 on mental health but also wellbeing. Additionally, this study recognized education qualification, employment type i.e. part-time or full-time and marital status were significantly associated with burnout which affected their mental health and wellbeing. Importantly, these associations were not recognized in a previous study conducted in Nepal [14]. This shows that when under graduates work as a part-time pharmacist, they tend to have difficulty while adapting skills, and may have uncertainty about their work which results in exhaustion and disengagement. In contrast, a study claimed that pharmacists with higher degrees were associated with severe psychological distress [15]. These findings underscore the importance of implementing workplace mentoring programs to better prepare pharmacy students, particularly during pandemics. Implementing occupational safety and health measures can help reduce psychological stress at the workplaces [25]. It is crucial for relevant organizations to review their services and allocate appropriate resources and support to address these issues effectively [28].

Limitations of the cross-sectional study include its inability to establish the direction and causality of the relationships between psychological health, burnout, and other variables. Furthermore, the study did not investigate the specific reasons why pharmacists were not taking sick leaves and considered leaving the pharmacy profession during the pandemic. Additionally, the study did not capture any changes in individual levels of burnout over time. The absence of pre-pandemic data restricts the ability to establish a clear understanding of any changes or trends in mental health indicators among pharmacists. Around 42% of sample were employed in community pharmacy, 38% in hospital pharmacy, and the rest were employed in industry, primary care, or academic institutions. The working conditions and the set of stressors in each one of these occupations is significantly different. A comparative analysis could have shed light on the specific effects of the pandemic on their mental health, including potential increases in stress levels, burnout, anxiety, depression, or other mental health challenges. Lastly, the time frame to recall the COVID-19 situation for the study group was from May 2021 to present which may have contributed to biases.

Future research may provide useful guidelines for pharmacists to prevent or reduce burnout in the pharmaceutical fraternity. Furthermore, added research on investigating what to do about exhaustion and disengagement and how to spot burnout and take action in time is required to control its negative impact on the mental health and wellbeing of pharmacists as well as other health professionals. It can help make the burnout tool a much more valid and reliable diagnostic measure to find out the extent of burnout affecting pharmacists.

The COVID-19 pandemic had a significant impact on the mental health and wellbeing of a sizable percentage of the pharmacists working as frontline health providers in Nepal. Burnout affected a noteworthy number of respondents associated with employment type i.e. part-time and undergraduate and postgraduate degrees. This study advocates for pharmacists to ensure their mental health and wellbeing in the workplace especially during these crucial times like pandemics as they have to be continuously serving public health. These findings clearly prove that many workplace cultures where pharmacists practice do not promote good mental health and wellbeing, which has a negative effect on productivity of the profession as a whole. This study provides valuable insights for developing targeted interventions and support systems. To address this issue, the government can consider implementing various measures like: promoting work-life balance for pharmacists, reducing excessive working hours, and developing strategies to effectively cope with challenges. Encourage organizations to adopt flexible work arrangements such as telecommuting. This allows working parents to adjust their work schedules to accommodate childcare responsibilities while fulfilling work obligations. Also offering subsidies for childcare services to alleviate the financial burden on families. Health policymakers must also consider a preventive approach to minimize the emergence of psychological symptoms. Additional measures to mitigate burnout may include emphasizing self-care practices, minimizing exposure to work-related stressors, encouraging open communication about workplace concerns, promoting regular physical exercise, maintaining personal hygiene, and ensuring a nutritious diet.

## Ethical approval

Human Research Ethics Committee of Thammasat University (Science), (HREC-TUSc); registration number: 117/2565. Nepal Health Research Council (NHRC); registration number: 528/2022 Kathmandu, Nepal.

## Informed consent statement

Informed consent was obtained from all participants involved in the study.

## Funding

Faculty of Public Health, Thammasat University, Thailand, contract no. 3/2566.

## Data availability

Q. Sharing research data helps other researchers evaluate your findings, build on your work and to increase trust in your article. We encourage all our authors to make as much of their data publicly available as reasonably possible. Please note that your response to the following questions regarding the public data availability and the reasons for potentially not making data available will be available alongside your article upon publication. Has data associated with your study been deposited into a publicly available repository?

Response: No.

Q. Sharing research data helps other researchers evaluate your findings, build on your work and to increase trust in your article. We encourage all our authors to make as much of their data publicly available as reasonably possible. Please note that your response to the following questions regarding the public data availability and the reasons for potentially not making data available will be available alongside your article upon publication. Has data associated with your study been deposited into a publicly available repository?

Response: Data included in article/supp. material/referenced in article.

## CRediT authorship contribution statement

**Subesha Adhikari:** Writing – review & editing, Writing – original draft, Validation, Methodology, Formal analysis, Data curation, Conceptualization. **Sarina Pradhan Kasaju:** Writing – review & editing, Methodology, Conceptualization. **Uma Langkulsen:** Writing – review & editing, Supervision, Methodology, Conceptualization.

## Declaration of competing interest

The authors declare the following financial interests/personal relationships which may be considered as potential competing interests: Uma Langkulsen reports financial support was provided by Faculty of Public Health, 10.13039/501100005790Thammasat University.
