# Supplementary material for: The influence of COVID-19 pandemic on the mental health of pharmacists as frontline health care providers in Nepal
Source: Heliyon. 2024 Apr 3;10(7):e29132. doi: 10.1016/j.heliyon.2024.e29132 (PMC11004875; doi:10.1016/j.heliyon.2024.e29132)
Supplement: Multimedia component 1 [file mmc1.docx]

**Note-**

**“Impact of work” pertains to negative impacts like- lack of breaks, staff shortages, increase demand for service and long working hours.**

**Mental health is defined as: “A state of emotional, psychological and social well-being in which an individual realizes their own abilities, can cope with the normal stresses of life, can work productively and is able to contribute to their community. It can be affected by things such as handling stress, making choices for one’s own self, etc.**

**Mental well-being is defined as, “The state of being comfortable, healthy, or happy”. It includes feeling good about one’s own self and about the decisions in life.**

***During COVID-19 pandemic includes the timeframe from May-June 2021 to Present**

**Questionnaire**

1. **Socio-demographic variables**
2. Age of the respondent
3. Sex
4. Female
5. Male
6. Others (Please specify)
7. Ethnicity:
8. Brahmin
9. Chhetri
10. Janjati
11. Ethnic minorities
12. Others (Please specify)
13. Religion:
14. Hindu
15. Buddhist
16. Christian
17. Muslim
18. Others (Please specify)
19. Education Qualification:
20. B. Pharm [ ]
21. Pharm D [ ]
22. MSc/M. Pharm [ ]
23. Post-graduate Pharmacy Fellowship [ ]
24. PhD [ ]
25. What is your main area of practice?
26. Community [ ]
27. Hospital [ ]
28. Academic/Educational body [ ]
29. Pharmaceutical industry [ ]
30. Primary care [ ]
31. At what stage of your career are you?
32. < 5 years [ ]
33. 5 – 10 years [ ]
34. >10 years [ ]
35. What is your employment type?
36. Part-time [ ]
37. Full-time [ ]
38. Marital status:
39. Single
40. Married
41. Divorced
42. Separated
43. Widowed
44. Family type:
45. Nuclear
46. Joint
47. Extended
48. **Mental health and well-being (Put a √ on each question below)**
49. During the COVID-19 pandemic which (if any) of the following have you experienced that you do not recall experiencing before the pandemic? (You can choose more than one of the choices)
50. Feeling down, depressed or hopeless [ ]
51. Trouble falling or staying asleep or sleeping too much [ ]
52. Poor appetite or overeating [ ]
53. Moving or speaking slowly that other people could have noticed or restless that you have been moving around a lot more than usual [ ]
54. Felt bad when things were not done ‘perfectly’ or a certain way [ ]
55. Felt symptoms in your body when you were worried or anxious (example tense muscles, stomach problems, headaches, fatigue) [ ]
56. Being afraid of feeling ill, having an illness or dying [ ]
57. Had repeated uncomfortable, thoughts and images that you found hard to ignore (example worries about contamination, worry that you are responsible for something bad happening) [ ]
58. Increased rate of cigarette smoking and drinking alcohol or taking more medications to feel better to calm yourself than you used to [ ]
59. If you checked off any of the complaints above, how difficult have these problems made it for you to work, take care of things at home, or get along with other people?
60. Not difficult at all [ ]
61. Somewhat difficult [ ]
62. Very difficult [ ]
63. Extremely difficult [ ]
64. Considering your responses to questions 11 and 12, how would you rate your overall mental health and wellbeing during the COVID-19 pandemic?
65. Very good [ ]
66. Good [ ]
67. Okay [ ]
68. Not good [ ]
69. Poor [ ]
70. During the COVID-19 pandemic, has your work had a positive or negative impact on your mental health and wellbeing?
71. Positive [ ]
72. Negative [ ]
73. Neither positive nor negative [ ]
74. Don't know/Not sure [ ]
75. Not applicable [ ]
76. Which of the following (if any) would you say have had a negative impact on your mental health and wellbeing? (You can choose more than one of the choices provided)
77. Financial problems [ ]
78. Finding full-time work [ ]
79. Illness [ ]
80. Inadequate staffing [ ]
81. Issues linked to studying/training [ ]
82. Lack of appropriate remuneration from employer [ ]
83. Lack of colleague or senior support on the job [ ]
84. Lack of work-life balance [ ]
85. Long working hours [ ]
86. Stress at work [ ]
87. Stress outside of work [ ]
88. Concerns about contracting COVID-19 or transmitting it to others [ ]
89. On a day-to-day basis, which of the following statements about work enjoyment best describes you?
90. I really enjoy my work [ ]
91. I enjoy my work [ ]
92. I am indifferent about my work [ ]
93. I don't enjoy my work [ ]
94. I really don't enjoy my work [ ]
95. Not applicable [ ]
96. During the COVID-19 pandemic, have you taken time off work (for sick leave or relieve exhaustion/disengagement) due to the impact of your work on your mental health and wellbeing?
97. Yes - a day to a few days in total [ ]
98. Yes - a week or more in total [ ]
99. Yes - a month or more in total [ ]
100. I have wanted to but have not felt able to [ ]
101. No [ ]
102. Don't know/Not sure [ ]
103. Not applicable [ ]
104. During the COVID-19 pandemic, how have you felt at the end of a typical working day?
105. Exhausted
106. Disengaged
107. Worried
108. Relaxed
109. Don’t know/Not sure
110. At any point during the COVID-19 pandemic, has the impact of your work on your mental health and wellbeing caused you to consider leaving your job or the pharmacy profession?
111. Yes, I have considered leaving the pharmacy profession [ ]
112. No, I have not considered this [ ]
113. Don't know/Not sure [ ]
114. Not applicable [ ]
115. During the COVID-19 pandemic, how frequently have you worried/do you worry about the quality of service you offer your service users, due to the impact of work on your mental health and wellbeing?
116. Always [ ]
117. Often [ ]
118. Sometimes [ ]
119. Occasionally [ ]
120. Never [ ]
121. Not applicable [ ]
122. During the COVID-19 pandemic, how frequently have you worried/do you worry about making mistakes in your work due to the impact of work on your mental health and wellbeing?
123. Always [ ]
124. Often [ ]
125. Sometimes [ ]
126. Occasionally [ ]
127. Never [ ]
128. Not applicable [ ]
129. **Oldenburg Burnout Inventory (Each question is answered on a Likert scale from 1 to 4, “strongly agree” to “strongly disagree” and reverse scoring for negative questions.**
130. **Strongly Agree**
131. **Agree**
132. **Disagree**
133. **Strongly disagree**

| **Items** | **1** | **2** | **3** | **4** |
| --- | --- | --- | --- | --- |
| 1. I often find new and interesting aspects in my work |  |  |  |  |
| 1. There are days when I feel tired before I arrive at work |  |  |  |  |
| 1. It happens more and more often that I talk about my work in a negative way |  |  |  |  |
| 1. After work I tend to need more time than in the past in order to relax and feel better |  |  |  |  |
| 1. I can tolerate the pressure of my work very well |  |  |  |  |
| 1. Lately I tend to think less at work and do my job almost mechanically |  |  |  |  |
| 1. I find my work to be a positive challenge |  |  |  |  |
| 1. During my work I often feel emotionally drained |  |  |  |  |
| 1. Over time I can become disconnected from my type of work |  |  |  |  |
| 1. After working I have enough energy for my leisure activities |  |  |  |  |
| 1. Sometimes I feel sickened by my work tasks |  |  |  |  |
| 1. After my work I usually feel worn out and weary |  |  |  |  |
| 1. This is the only type of work that I can imagine myself doing |  |  |  |  |
| 1. Usually, I can manage the amount of my work well |  |  |  |  |
| 1. I feel more and more engaged with my work |  |  |  |  |
| 1. When I work I usually feel energized |  |  |  |  |
